# Lithuania’s National Digital Health System Architecture, Evolution, and Lessons From a Centralized Approach Using Longitudinal Document Analysis: Descriptive National Case Study

**DOI:** 10.2196/91911

**Published:** 2026-09-02

**Authors:** Austėja Dapkutė, Olegas Niakšu, Julius Juodakis, Danguolė Jankauskienė, Justas Trinkūnas, Arvydas Laurinavičius, Tomas Jovaiša, Donata Ringaitienė, Martyna Atraškevičienė, Rytis Masiliūnas

**Affiliations:** 1 Clinic of Neurology and Neurosurgery, Institute of Clinical Medicine Faculty of Medicine Vilnius University Vilnius Lithuania; 2 Center for Digital Medicine, Institute of Translational Health Research Faculty of Medicine Vilnius University Vilnius Lithuania; 3 State Data Agency Vilnius Lithuania; 4 Health Research Laboratory Mykolas Romeris University Vilnius Lithuania; 5 Department of Information Systems Faculty of Fundamental Sciences Vilnius Gediminas Technical University Vilnius Lithuania; 6 Clinic of Anaesthesiology and Intensive Care, Institute of Clinical Medicine Faculty of Medicine Vilnius University Vilnius Lithuania; 7 Innovation and Technology Transfer Department Vilnius University Hospital Santaros Klinikos Vilnius Lithuania

**Keywords:** digital health platform, digital health, eHealth, electronic health records, health information systems

## Abstract

**Background:**

National digital health systems are increasingly recognized as a key tool for strengthening health care systems and improving patient outcomes. Global guidance from the World Health Organization and European Union initiatives emphasizes interoperability, robust data governance, and secure exchange of health information as prerequisites for system-wide benefits. However, translating these principles into operational national digital health infrastructures remains challenging, and empirical evidence on long-term, centralized implementation models is limited. Lithuania is among a small number of European countries that have implemented a fully centralized national digital health platform (DHP), providing a valuable case for examining the development, structure, and performance of such systems.

**Objective:**

This study aimed to describe and analyze Lithuania’s national digital health system across its historical development, governance, technical architecture, interoperability and adoption, and to identify challenges and lessons learned.

**Methods:**

We conducted a descriptive national case study of Lithuania’s digital health system using longitudinal document analysis. Publicly available policy documents, legislation, strategy reports, audits, and official statistics published between 2000 and 2025 were systematically reviewed following the readying, extracting, analyzing, and distilling (READ) framework. Thematic analysis was applied to examine governance, system architecture, interoperability, adoption, and implementation challenges. Peer-reviewed literature was used for contextualization.

**Results:**

Lithuania’s digital health system evolved from fragmented initiatives in the early 2000s into a fully centralized national DHP that has been operational nationwide since 2015. Since 2018, health care providers have been required to record core clinical data electronically, and all reimbursed medicines are prescribed exclusively through the national electronic prescription system. The DHP integrates electronic health records, electronic prescription system, appointment booking, and other national subsystems with standardized interfaces. System use expanded rapidly: the annual number of electronic documents stored in the DHP increased from approximately 7 million in 2017 to over 100 million in 2024, reflecting widespread uptake of outpatient documentation, referrals, and prescriptions. Patient-facing services, including online access to records and a nationwide appointment booking system, also expanded, although patient-initiated use remains uneven across regions. The platform supports secure primary use for care delivery and secondary use of health data through a centralized access pathway. While core services have reached national scale, some subsystems, such as imaging exchange and telemedicine, remain less mature. Ongoing modernization focuses on improving data interoperability, enabling advanced analytics and future AI-based services, strengthening cross-border eHealth services, and extending coverage to sensitive and previously excluded domains such as mental health, maternal, and newborn care through dedicated subsystems.

**Conclusions:**

Lithuania’s centralized digital health system illustrates both the potential and the limitations of national-scale integration driven by legal mandates and standardized interoperability. Its experience provides transferable insights for health systems seeking to consolidate fragmented infrastructures while maintaining data security, regulatory compliance, and long-term system adaptability.

## Introduction

The implementation of digital health solutions has been actively promoted by global and regional health organizations, including the World Health Organization (WHO) Resolution WHA58.28 [1,2], which recognized eHealth as a key tool for strengthening health care systems and improving patient outcomes. In the European Union (EU), digital health policies are shaped by the European Health Data Space (EHDS) initiative [3] and the General Data Protection Regulation (GDPR) [4], which enforce secure, interoperable, and patient-centered digital health services across member states, facilitate cross-border health care access, and enable efficient exchange of health data. In this paper, “digital health” is used as the primary term to describe the broader ecosystem of health information technologies and services. The term “eHealth” is retained when referring to historical policy frameworks, national program names, and terminology used in official documents.

A structured national digital health system enhances communication between health care providers, optimizes service efficiency, and supports evidence-based decision-making. According to the WHO Global Strategy on Digital Health (2020-2025), the key elements of a successful digital health framework include interoperability, robust data governance, scalability, and patient-oriented digital services such as electronic health records (EHRs) and telemedicine solutions [5]. Ensuring the smooth implementation of these systems, however, remains a complex task. Therefore, it is crucial for countries to exchange insights into how they implement these key elements and address the challenges they encounter.

Lithuania is a Baltic state, located in Northern Europe, with a population of 2.89 million and an area of 65,300 km^2^ [6]. In 2024, life expectancy was 77.6 years, 4.1 years below the EU average, with a marked gender gap (81.9 years for women vs 73.1 years for men) [7]. Cardiovascular diseases remain the leading cause of death, accounting for 53% of all deaths in 2023, while cancer accounted for 21% [7]. Lithuania’s health system is financed predominantly through compulsory statutory health insurance under a single-payer structure centered on the National Health Insurance Fund (NHIF) and its territorial funds, which administer the Compulsory Health Insurance Fund and reimburse covered services, medicines, and medical aids for insured residents [8]. Total health expenditure was 7.3% of the gross domestic product (GDP) in 2023, equivalent to EUR 2397 per capita, with public funding accounting for 68% of total health spending [7].

According to the EU Digital Decade Policy Program 2030 assessment, Lithuania is among the top 4 countries providing citizens access to EHRs (98% maturity in 2026) [9]. Named a “trendsetter” [9], Lithuania collects national-coverage health care data in 2 large databases of health records: the nationwide digital health platform (DHP), managed by the State Enterprise Centre of Registers (SECR), and the health insurance system SVEIDRA (Lithuanian title of a National Health Insurance System), managed by the NHIF [10]. The DHP includes essential health data, such as referrals, inpatient/outpatient visit summaries, and electronic prescriptions (ePrescriptions), from both public and private health institutions providing primary, secondary, and tertiary care, whereas SVEIDRA records and monitors medical services reimbursed through the Compulsory Health Insurance Fund, encompassing outpatient and inpatient services, reimbursed medicines, and related administrative processes.

In addition, several smaller registers and hospital information systems (HIS) also contain important EHRs that are not shared with the DHP or SVEIDRA and remain distributed throughout the health care system. All these systems can be accessed and linked by the country’s state data agency (SDA), a governmental institution responsible for national statistics and data governance [10,11]. However, challenges remain in data interoperability, interinstitutional collaboration, and cross-border health data exchange, requiring further integration with EU-wide initiatives, including the EHDS, which is expected to facilitate data sharing between EU health care systems.

Lithuania is one of the few European countries with a fully centralized digital health approach and provides a useful case for examining the development and implementation of such systems. This case study aims to provide an overview of Lithuania’s digital health architecture, examining its evolution, core components, achievements, and ongoing challenges.

## Methods

### Case Study Approach

We conducted a descriptive and interpretative case study of Lithuania’s national digital health system to analyze its evolution, governance, infrastructure, and challenges. This approach allows for a detailed, context-sensitive understanding of a complex policy implementation process, similar to methods used in political science and public policy analysis [12]. To capture evolution across policy cycles and technological phases, we adopted a longitudinal perspective, examining documents and policies spanning from 2000 to 2025. This study used only publicly available documents and involved no human participants; therefore, institutional ethical approval and informed consent were not required.

### Data Sources and Collection

We followed the readying, extracting, analyzing, and distilling (READ) approach to guide our document review and ensure a systematic and transparent process [13]. This approach conceptualizes documents as informants, such as qualitative interviews, allowing us to extract and interpret key policy themes and stakeholder perspectives from official texts.

**Figure 1:**
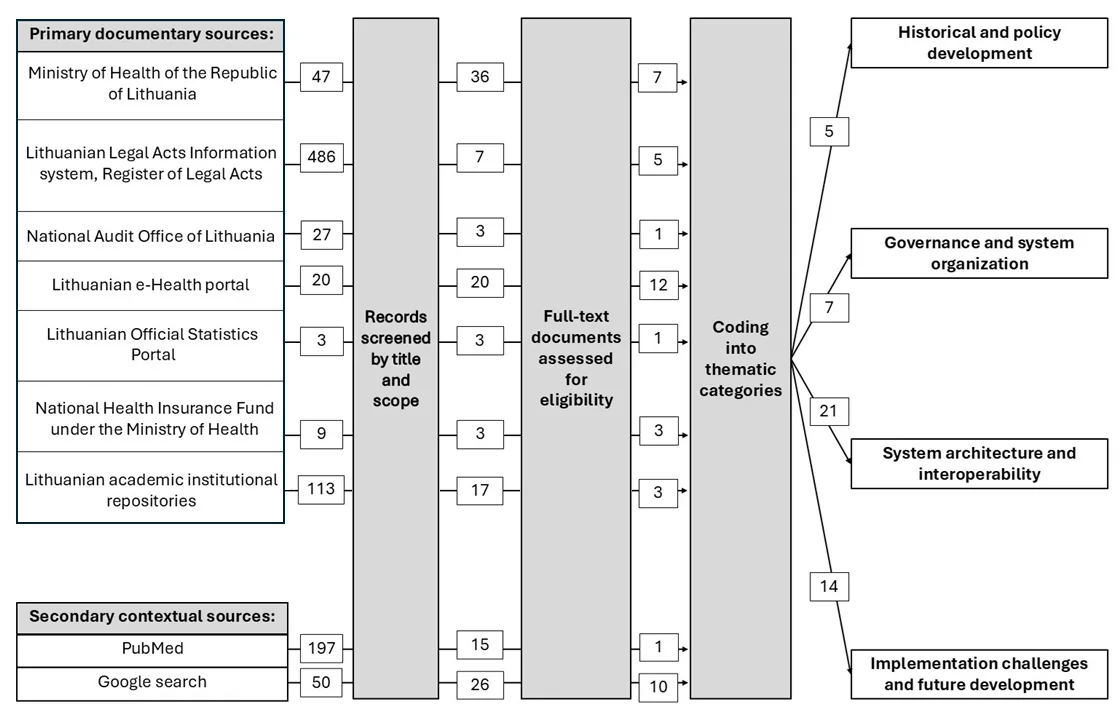
Flowchart of identification, screening, and selection of primary documentary sources and secondary contextual literature for the analysis of Lithuania’s digital health system.

Between February 2025 and June 2025, we conducted a structured search and collection of publicly available documents related to the development, governance, and implementation of Lithuania’s digital health system. Primary documentary sources included policy documents, legal acts, strategic plans, ministerial orders, institutional reports, and official announcements published by Lithuanian governmental and public-sector bodies. Key sources comprised the Ministry of Health of the Republic of Lithuania, the Register of Legal Acts, the Lithuanian eHealth portal [14], the Official Statistics Portal of Lithuania, the NHIF, the National Audit Office of Lithuania, and selected Lithuanian academic institutional repositories (Figure 1 [14-23]). Documents were reviewed in Lithuanian and English.

Document identification was conducted in stages. First, targeted searches were performed within institutional sources using Lithuanian keywords such as “ESPBI” (the national DHP system acronym) and “elektroninė sveikata” (Lithuanian term for electronic health). Second, Google was used as a supplementary search tool, applying combinations of Lithuanian and English keywords (“elektroninė sveikata,” “ESPBI,” “Lithuania” with “eHealth” or “digital health”). Given the large number of results, a subset of top-ranked results (n=50) was screened for relevance. Third, PubMed was used to identify relevant peer-reviewed publications using the query “Lithuania” AND (“electronic health record” OR “health information system” OR “eHealth” OR “digital health”) for publications from 2015 onward.

Identified records were screened by title and scope, and relevant documents were selected for full-text review. The selection process is summarized in Figure 1. Following selection, documents were organized into a structured matrix by publication year, document type, and thematic focus. Content analysis was conducted iteratively in several stages. Documents were reviewed by at least 3 authors, and key information was extracted and compared across sources. Emerging themes were identified and refined through structured discussions among the author team, leading to the development of higher-level thematic categories. Consensus on interpretation was achieved through iterative comparison of sources and group discussion, with designated authors coordinating specific thematic areas.

The analysis also incorporated the authors’ domain expertise. The author team includes individuals with professional experience in digital health policy, implementation, and evaluation within Lithuania. This perspective was applied reflexively to interpret findings, clarify institutional processes, and triangulate documentary evidence, while maintaining primary reliance on documented sources. Triangulation was achieved through comparison of information across multiple documentary sources, including policy documents, legislation, audits, institutional reports, official statistics, and peer-reviewed literature.

### Inclusion and Exclusion Criteria

We included documents that explicitly addressed the development, governance, or implementation of Lithuania’s digital health architecture at the national level. This included strategic plans, legal acts, technical reports, and official evaluations related to EHRs, health data governance, and interoperability.

We excluded documents that focused solely on institutional-level initiatives without national relevance, materials unrelated to health system digitalization, and documents with only tangential mentions of digital health (eg, general health policy without digital components).

### Analysis

We applied thematic document analysis to the collected materials. The analysis followed an iterative, inductive process involving reading each document by at least two authors, extraction of relevant information into a structured evidence matrix, comparison across sources, and synthesis into higher-level thematic categories. Themes were refined through discussion among the authors and organized around historical development, governance, system architecture, interoperability, adoption, and implementation challenges. Quantitative indicators obtained from official statistics and administrative reports were extracted and summarized descriptively to illustrate trends in system use, document coverage, and national adoption.

### Ethical Considerations

This study was based exclusively on the analysis of publicly available documents and did not involve human participants, personal data, or identifiable individual-level information. Therefore, it does not fall under the scope of biomedical research involving human subjects as defined in the Lithuanian Law on Biomedical Research Ethics [24], and ethical review board approval was not required.

## Results

### Historical Milestones

**Figure 2:**
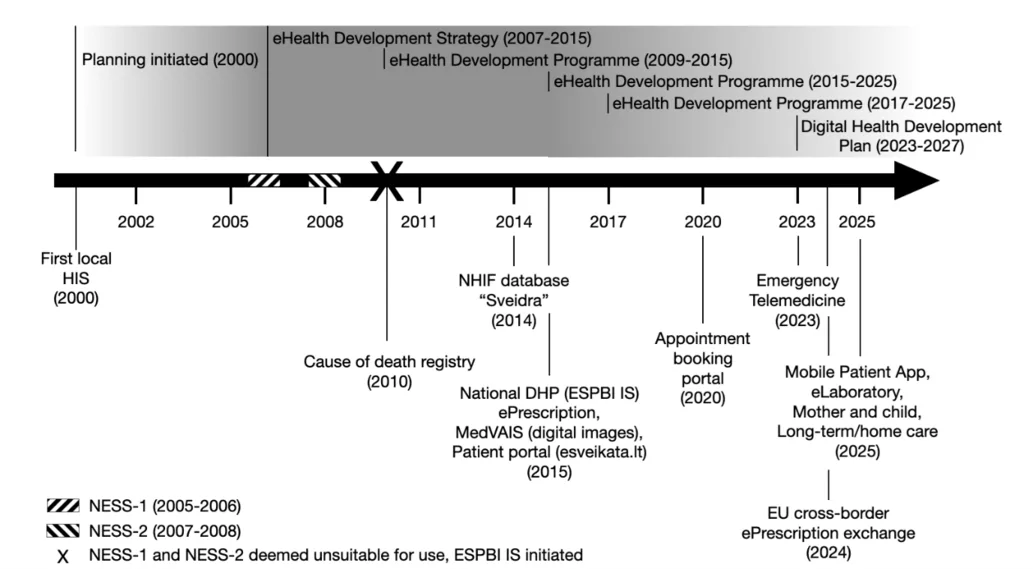
Lithuanian eHealth development milestones. DHP: digital health platform; Emergency Telemedicine: hub-and-spoke telemedicine between Emergency Departments; ESPBI IS: Electronic Health Services and Cooperation Infrastructure Information System (Elektroninė sveikatos paslaugų ir bendradarbiavimo infrastruktūros informacinė sistema); EU: European Union; HIS: hospital information system; NESS: National eHealth Service System; NHIF: National Health Insurance Fund.

The development of Lithuania’s health information system began in the early 2000s. However, integration into the EU in 2004 triggered a strategic reassessment of its digital health infrastructure. This led the Ministry of Health to conduct a comprehensive analysis of the existing digitalization framework. This analysis led to a feasibility study, which subsequently informed funding and the development of a comprehensive eHealth system implementation roadmap [25]. The initial deployment of the National eHealth Service System (NESS) commenced in 2005, marking a significant milestone in Lithuania’s digital health initiative (Figure 2). The first phase, termed NESS-1, spanned from 2005 to 2007 and focused on the first attempt to establish the National eHealth Core with essential tools for information exchange. Its objectives subsequently included implementing several pilot functions of the national-level eHealth system, deploying them across regional health care institutions, and automating relevant internal processes [26]. It was funded with World Bank and EU structural funds [27]. NESS-1 was supposed to include the development of HIS for the 3 largest regional hospitals in Lithuania and a common centralized DHP [26].

This was succeeded by the second phase, NESS-2, from 2007 to 2008. During this phase, HIS were implemented in the 3 largest university hospitals and integrated with a pilot health information exchange backbone based on Integrating the Healthcare Enterprise (IHE) profiles [28,29], as defined by the EU epSOS (Smart Open Services for European Patients) project [30]. However, institutional governance was fragmented throughout much of this period. To address this, the eHealth Development Coordination Board was established in 2009, bringing together representatives of the Ministry of Health, major hospitals, and experts in IT and health policy [31]. Later, more stakeholders were involved, such as representatives of regional hospitals, physician associations, and patient organizations. It was established as an advisory organ to coordinate the preparation and implementation of the eHealth development strategy and its implementation plan, assess the compatibility of all eHealth-related projects that apply for funding, and evaluate their feasibility and cost [25]. The Health System Information Resources Development Division within the Ministry of Health was responsible for operational management of digital health initiatives, overseeing the planning, coordination, and supervision of health information system development. The division ensures alignment with national standards and legal frameworks, manages data governance activities, and supports the implementation and monitoring of digital health projects across the sector. However, the initial NESS I and NESS II programs did not achieve their intended objectives. Following an evaluation conducted by the Ministry of Health in 2010, the programs were discontinued and replaced with a new centralized architecture. Subsequent audits identified a combination of strategic planning deficiencies, fragmented coordination, repeated implementation delays, incomplete functionality, limited user adoption, and insufficient integration as key factors contributing to the discontinuation and restructuring of the original approach [32]. The Lithuanian nationwide DHP was developed during 2012-2015 [32,33]. It included 13 regional and 16 national projects, concentrating on 4 core systems: the health information exchange platform “*Elektroninė sveikatos paslaugų ir bendradarbiavimo infrastruktūros*” (ESPBI), patient and specialist portals (national ePrescription), and national picture archiving and communication systems (PACS) “MedVAIS,” along with 6 minor projects and various HIS (Figure 2), that encompassed initial steps toward the digitalization of medical documentation [32]. In 2015, the nationwide DHP became accessible to patients and health care professionals [34]. The strategic development continued with the Lithuanian eHealth system development program from 2015 to 2025, aimed at further enhancing the robustness and scope of digital health services [32].

In 2017, the National Audit Office evaluated Lithuania’s eHealth development and identified a series of systemic weaknesses [32]. The audit found fragmented governance, with uncoordinated targets across planning documents and a weak results framework characterized by insufficient measurable indicators and unclear methodologies. Funding decisions were often project-driven without provisions for sustainable maintenance. Earlier design errors were repeated, including the lack of mechanisms to encourage or enforce system use and the introduction of unjustified telemedicine solutions. Project management and coordination were inadequate, with no integrated implementation plan and repeatedly extended deadlines. Systems were frequently accepted into operation without full functionality, proper quality assurance, or complete documentation, while duplication of regional solutions led to irrational spending. Uptake remained low, as many providers did not submit data, a significant proportion of pharmacies were unprepared, and patient portal registration rates were modest. Integration interfaces were incomplete or nonfunctioning, incident management and system maintenance arrangements were immature, and controls over e-signature use were insufficient. Finally, organizational and technical measures to protect sensitive personal data were found to be inadequate [32]. Although considered flawed, early NESS projects were important as the first national-level digital health initiatives, which brought interoperability and introduced the key industry standards such as Health Level 7 (HL7) and Logical Observation Identifiers Names and Codes (LOINC), serving as a base for further digital health development in the form of the national DHP.

Since 2018, all data related to patient diagnostics, treatment, and referrals have been required to be managed via DHP and stored electronically [35,36]. This has encouraged a further eHealth development program for 2017-2025 [37], aiming to increase the safety of personal data, to modernize and enhance ePrescription usage, and to increase the number of users of the national DHP [35], which has peaked dramatically over recent years. In 2024, 89.1% of Lithuanians aged 16-74 years used information technologies, with 55.4% accessing the EHRs and 47.1% using online services to schedule doctor’s appointments [38]. Transparency is further strengthened through online public sources, such as the appointment booking system waiting-time dashboard, which publishes and compares appointment queues across providers [39] and a national quality dashboard with indicators such as rate of hospitalization with chronic conditions, percentage of used hospital beds and rate of surgical complications [39], that allow hospitals, policymakers and patients to compare key aspects of service quality between health care institutions.

**Figure 3:**
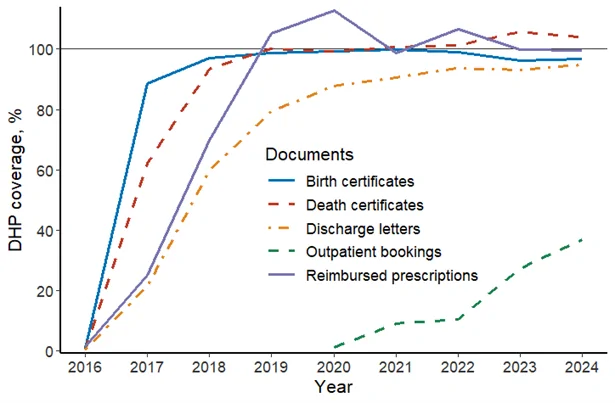
Approximate share of selected medical documents recorded in the national digital health platform (DHP), 2016-2024. Coverage is calculated as the ratio of documents stored in the DHP to national reference sources: births in the DHP vs official statistics of births, deaths in the DHP vs official statistics of deaths of permanent Lithuanian residents, inpatient stays vs number of stays reimbursed by the national insurance (National Health Insurance Fund [NHIF]), outpatient bookings via DHP vs number of reimbursed nonemergency outpatient visits, and amount spent on reimbursed prescriptions in the DHP vs corresponding amount from the NHIF. The estimates are approximate and may exceed 100% due to differences in numerator and denominator definitions, data collection processes, and timing. For example, DHP data may include services not reimbursed by NHIF or recorded under different reporting cycles; population-based statistics (eg, births and deaths) may use residency-based definitions that differ from health care system records. In addition, delays in reporting and retrospective data updates may lead to temporary discrepancies between sources.

The total number of electronic documents stored in the DHP per year rose from nearly 7 million in 2017 to 109 million in 2024 (Figure 3 and Table 1). Key document types included 37 million forms for outpatient visits, 22 million ePrescriptions, 10 million specialist referrals, 2 million responses to referrals, and 1 million inpatient discharge notes issued online in 2024. Other digital documents include routine medical check-up notes for drivers, children, and workers, vaccination notes, and death certificates. The number of medical images described online also rose significantly, from 0.27 million in 2018 to 2.81 million in 2024 per year. As of 2025, 663 health care institutions offer electronic registration services, involving 2046 general practitioners and 7102 specialists. With a dedicated appointment booking system being launched in 2020, patient-initiated electronic registrations for visits have expanded from 18,000 in the first quarter of 2022 to 72,000 in the first quarter of 2025 for general practitioners and from 93,000 to 206,000 for specialists over the same period [40]. With the implementation of cross-border EU/European Economic Area ePrescription services, a total of 497 prescriptions were dispensed between May 2024 and May 2025 in Lithuania for patients from abroad, primarily Latvia (n=449) and Estonia (n=23), while during the same period, 625 Lithuanian ePrescriptions were serviced in other countries, mainly in neighboring Poland (n=336) and Latvia (n=222) [40].

**Table 1:** Trends of the digital health platform usage in Lithuania. Number of electronic documents signed, 2016-2024 [1,2].

Description	2016	2017	2018	2019	2020	2021	2022	2023	2024
Outpatient visits (millions)	0.5	1.4	5.0	10.0	14.9	27.1	27.6	32.3	36.5
Reimbursed prescriptions (millions)	0.2	2.8	9.1	12.8	15.6	16.1	18.4	19.7	21.7
Referrals (millions)	0.0	0.0	0.3	1.5	3.7	6.7	8.0	9.4	10.1
Discharge letters (millions)	0.0	0.2	0.4	0.6	0.5	0.5	0.6	0.6	0.7
Birth certificates (thousands)	0.3	24.7	26.0	24.7	23.4	23.3	21.8	19.8	18.5
Death certificates (thousands)	0.1	25.0	37.0	38.3	43.2	48.1	43.4	39.2	39.0
Medical image descriptions (millions)	0.0	0.1	0.3	0.7	1.0	1.8	2.1	2.5	2.8
Medical images uploaded (millions)	0.1	0.1	1.3	1.8	1.5	1.8	1.8	1.4	1.6
Outpatient bookings (millions)	—^a^	—	—	—	0.3	2.8	3.6	9.6	13.9

^a^Not available.

### Lithuanian Digital Health Ecosystem Architecture

#### Overview

Historically, Lithuania’s health reimbursement data have been collected by NHIF, which manages the Compulsory Health Insurance Fund and oversees payments for health care services and reimbursable medicines, as well as budgeting, auditing, and pricing functions [41]. Before the establishment of the national DHP, these reimbursement records represented the main source of national health statistics, as no other comprehensive data collection existed. Statistical functions were formally the responsibility of the Institute of Hygiene and later also the SDA, which currently produces official statistics using data from national registers, including NHIF.

**Figure 4:**
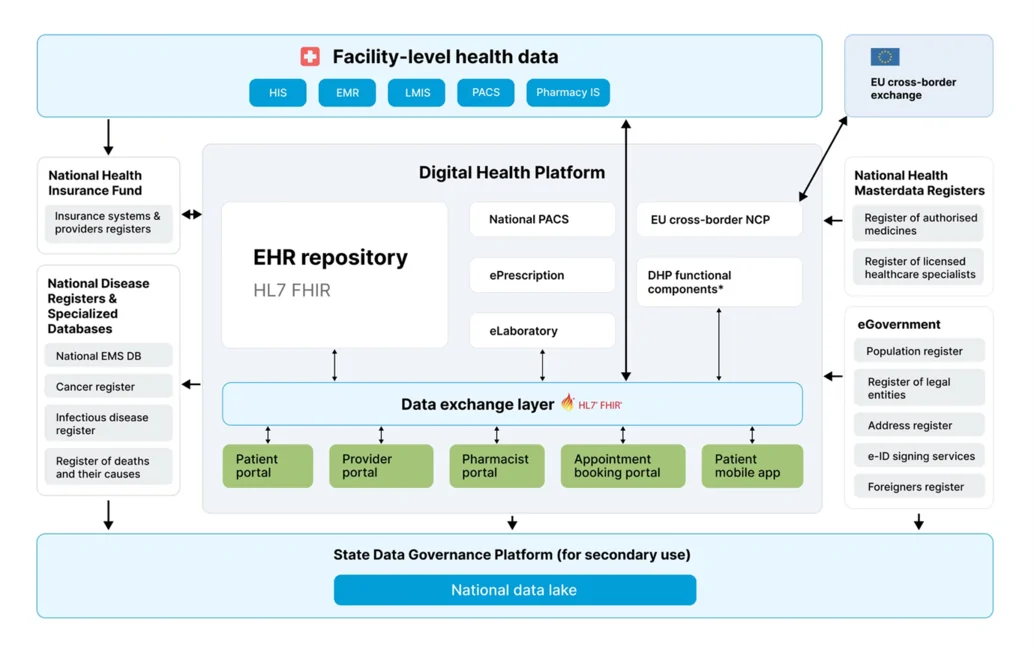
Model of the Lithuanian digital health ecosystem. DB: database; DHP: digital health platform; EHR: electronic health record; EMR: emergency medicine records; EMS: emergency medical services; EU: European Union; FHIR: Fast Health Interoperability Resources; HIS: hospital information system; HL7: Health Level 7; IS: information system; LMIS: laboratory medicine information system; NCP: National Contact Point; PACS: picture archiving and communication system.

Upon the creation of the centralized DHP, the central role for its management was allocated to SECR. It is also in charge of the operation of the key national registers and cadastre. Currently, the Lithuanian digital health ecosystem is composed of the centralized DHP, national information systems/registers, and local systems, such as emergency medicine registers, HIS, laboratory medicine information systems, and PACS (Figure 4 and Table 2). Health facilities are legally required to transmit the defined patient health data from the facility-level systems to the DHP, where it is combined with data from other systems, such as data on specialist and facility licenses. Interoperability between local HIS and the DHP is mandated in national regulation: providers must submit clinical documents using nationally defined message and document templates, based on HL7 Fast Healthcare Interoperability Resources (FHIR) format [42]. In routine operation, event-level records are transmitted to the DHP in near real-time (or promptly after service restoration), whereas reimbursement-related data, such as claims datasets, are submitted to the NHIF system on scheduled reporting cycles and therefore update more slowly. The clinical documents submitted to the DHP are signed using qualified electronic signatures, which ensure their legal validity, integrity, and nonrepudiation. Access to DHP data is strictly role-based and audited. Multiple identification and authentication methods with a high level of assurance are supported; patients and health professionals are authenticated via national eGovernment identification services.

**Table 2:** Main systems and components of the Lithuanian digital health ecosystem.

Component		Manager	Function
**Core eHealth systems**			
	EHR^a^ repository (ESPBI IS^b^)	SECR^c^	Acts as a national EHR and health information exchange platform, which centralizes and integrates national patient health data for secure access and exchange across health care institutions. It also stores clinical documents, such as discharge letters, referrals, consultation notes, and certificates, providing the backbone for patient record continuity.
**DHP^d^ components**			
	ePrescription^e^	SECR	Enables electronic creation and management of prescriptions and recording of dispensations.
	National PACS^f^—MedVAIS	SECR	Acts as a national PACS, which archives and enables exchange of diagnostic imaging data across institutions.
	National eLaboratory—eLAB	SECR	Manages electronic lab test orders and lab results in a structured and standardized way.
	National Remote Purchasing of Prescription Medications System—NPRVP^g^	SECR	Enables direct online purchase of prescription medicines and medical aids from eligible online pharmacies.
	The electronic drug interaction system	SECR	Checks medication compatibility and alerts providers to potential drug interactions during prescribing.
	Appointment booking portal	SECR	Provides nationwide online appointment booking capacity for primary and specialized outpatient care.
	Patient portal, specialist portal, pharmacist portal, and patient mobile app	SECR	Provide role-specific portals for patients, health professionals, and pharmacists to access and manage health data.
**Health-related registers and monitoring systems**			
	Hospital information systems	Multiple	Manage clinical and administrative data at the institutional level
	Register of licensed pharmacists and assistant pharmacists (part of VAPRIS^h^)	SMCA^i^	Stores and verifies credentials of licensed pharmacists and assistant pharmacists.
	National Register of Authorized Medicines (part of VAPRIS)	SMCA	Lists authorized pharmaceuticals with detailed product, usage, and regulatory information.
	Adverse event reporting system (part of VAPRIS)	SMCA	Enables reporting of suspected adverse reactions to medications and vaccines.
	Register of blood donors	IH^j^	Stores donor identity, donation history, and test results.
	Register of deaths and their causes	IH	Registers all deaths and causes nationally, including Lithuanians deceased abroad, for mortality statistics and analysis.
	Register of public health specialists	IH	Registers and manages data on professionals performing public health functions in state and municipal institutions.
	Children health monitoring IS^k^	IH	Collects and analyses health data of schoolchildren.
	Public health monitoring IS	IH	Aggregates and processes public health statistics.
	Injury and accident monitoring system	IH	Centralizes data on injuries and accidents.
	State information system for infectious diseases and their pathogens	NPHC^l^	Collects, stores and analyses data on communicable diseases and their pathogens to support epidemiological surveillance, outbreak investigation and prevention in Lithuania.
	Emergency management information system (ESVIS^m^)	MoH^n^	Manages and monitors health emergencies (eg, epidemics, bioterrorism, chemical incidents) and coordinates health care providers and the national health system during extreme health events.
	Medical nomenclatures and classifiers management IS	LLM^o^	Provides health care professionals and laboratories with access to, and management tools for, standardized clinical terminologies and laboratory test nomenclatures (such as SNOMED CT^p^, LOINC^q^ and the national laboratory test nomenclature).
	Register of licensed health care specialists	VASPVT^r^	Tracks and verifies credentials of licensed medical professionals.
	Register of licensed health care legal entities	VASPVT	Maintains licensing data for health care institutions to ensure regulatory compliance.
	Cancer register	NCI^s^	Collects nationwide cancer incidence, mortality, and survival data for monitoring and research.
	Queue management information system	NHIF^t^	Manages waiting lists and resource allocation for insured medical aids, devices, and treatments.
	SVEIDRA^u^	NHIF	Accounts for and monitors reimbursed medical services under Lithuania’s health insurance system, allows verification of the validity of individual compulsory health insurance status.
	Electronic certificates management system	SODRA^v^	Enables issuance of electronic sick leave and maternity certificates for social benefit processing.
**Other national systems and registers**			
	Population register	SECR	Provides demographic data to support accurate patient identification.
	Address register	SECR	Provides official address data to support location accuracy in health care services and emergency planning.
	Register of legal entities	SECR	Maintains official data on organizations, supporting provider verification and compliance in health care.
	SDG IS^w^	SDA^x^	Collects, stores, processes, and integrates data from state information systems.

^a^EHR: electronic health record.

^b^ESPBI IS: Lithuanian digital health platform “Elektroninė sveikatos paslaugų ir bendradarbiavimo infrastruktūros informacinė Sistema.”

^c^SECR: State Enterprise Centre of Register.

^d^DHP: digital health platform.

^e^ePrescription: electronic prescription.

^f^PACS: picture archiving and communication system.

^g^NPRVP: Nuotolinės prekybos receptiniais vaistiniais preparatais ir MPP posistemė.

^h^VAPRIS: Lithuanian title for electronic system of State Medicines Control Agency.

^i^SMCA: State Medicines Control Agency.

^j^IH: Institute of Hygiene.

^k^IS: information system.

^l^NPHC: National Public Health Centre.

^m^ESVIS: Emergency Management Information System.

^n^MoH: Ministry of Health.

^o^LLM: Lithuanian Library of Medicine.

^p^SNOMED CT: Systematized Nomenclature of Medicine-Clinical Terms.

^q^LOINC: Logical Observation Identifiers Names and Codes.

^r^VASPVT: State Accreditation Service for Health Care Activities.

^s^NCI: National Cancer Institute.

^t^NHIF: National Health Insurance Fund.

^u^SVEIDRA: Lithuanian title of a national health insurance system.

^v^SODRA: State Social Insurance Fund Board.

^w^SDG IS: state data governance information system/state data lake.

^x^SDA: state data agency.

The DHP provides means for patients and health professionals to access and create health records through dedicated patient and specialist portals. Although the obligation to record patient data in the DHP has been applied since 2018, mental health records were deferred pending appropriate safeguards. In 2026, a dedicated mental health subsystem is planned to be added, bringing these records into the DHP with enhanced protections: full visibility only for psychiatrists, with limited views for family doctors (diagnosis, investigations, treatment, and risk factors) [43]. This integration ensures that health information is interoperable, consistently updated, and accessible across care levels. In late 2025, a new home nursing subsystem [44] and a subsystem for pregnant women were introduced [45]. The latter is planned to be supplemented with data on women in labor and newborns starting in mid-2026 [45].

Consolidated health data are made available for secondary use via the SDA, Lithuania’s national statistics authority. Since the 2022 Law for Health Data Reuse, the SDA operates a single access pathway and a secure remote analysis environment (or data lake) through which datasets from the DHP and other state registers can be linked and analyzed. Only disclosure-checked outputs may leave the environment. This arrangement enables population-level monitoring, research, and innovation under a common governance, access, and audit framework [10,11].

#### Facility-Level Information Systems

In Lithuania, health care institutions use a range of local information systems developed by multiple private providers. Notably, Vilnius University Hospital Santaros Klinikos has been a pioneer in the digitalization of health data and administrative processes. Their in-house developed Santa HIS system, introduced in 2000, supported inpatient care in a tertiary setting and has since been implemented in several other hospitals. Other tertiary hospitals introduced HIS solutions around 2008, and by 2015 all regional and district hospitals had adopted HIS. Larger public and private outpatient clinics typically use commercial EHR systems [32], while smaller providers rely on the free national providers’ portal made available through the DHP. Furthermore, specific domains within health care are addressed by specialized independent systems such as outpatient clinical information systems [46].

Although the DHP was developed as a central platform to support clinical documentation, data exchange, and access to patient health records, its practical implementation has encountered some challenges. Users have noted difficulties related to system performance, interface usability, and information retrieval, which have impacted daily operations in certain settings. At the same time, the DHP continues to serve as a national data exchange platform and, for many smaller providers, as a basic solution for managing core clinical datasets. Many health care institutions, particularly larger hospitals, also rely on or invest in local HIS that are tailored to their specific clinical workflows and offer more intuitive interfaces, while integrating with the DHP and reusing centrally provided components such as ePrescription and other subsystems. Some of these local systems have evolved rapidly, offering functionalities that complement or, in some cases, exceed those of the central platform in specific areas.

#### The Core eHealth Information System—DHP

##### Overview

At the center of Lithuania’s national digital health infrastructure is the DHP, formally known as the Electronic Health Services and Cooperation Infrastructure Information System (*Elektroninė sveikatos paslaugų ir bendradarbiavimo infrastruktūros informacinė sistema* [ESPBI IS]) and managed by SECR. The DHP development started in 2011 [32], and the system has been widely accessible in routine practice since 2015, integrating data from facility-level systems and used by all health care providers in Lithuania [36]. Data are transferred to the DHP either automatically or semiautomatically, often requiring qualified e-signatures, and subsequently, data are used for primary use by clinicians and are transferred for secondary use to other national registers and information systems.

The core platform is divided into 3 primary components: a data exchange layer, a national EHR repository, and an application layer composed of user portals. The data exchange layer consists of web services and an HL7 FHIR API that facilitate data exchange between health care institutions’ systems and the central platform. The underlying data are stored in an Oracle relational database management system using Structured Query Language. Clinical documents are exchanged typically as XML-based FHIR compositions. In addition to structured data, noneditable and digitally signed PDF versions of the same clinical documents are archived to ensure legal validity and auditability. The system uses encryption, access controls, and audit logs to protect sensitive health information while enabling authorized data sharing between institutions and sectors. It supports EU cross-border eHealth services, such as exchange of ePrescriptions. From a legal data governance perspective, to participate in data exchange with the DHP, health care providers are required to establish data exchange agreements with SECR. Institutions that fail to integrate are subject to regulatory oversight and potential sanctions, emphasizing the critical role of the DHP in national health care delivery [47].

The DHP aims to integrate all patient health information, including medical histories, laboratory test results, diagnostic data, prescribed medications, and referrals, and form a longitudinal health record. This approach is enhanced by DHP subsystems such as the ePrescription [48] and medical imaging (national PACS) modules [49], enabling health care providers to access and manage patients’ prescriptions and diagnostic imaging. Telemedicine services, currently focused on emergency medicine and aimed to cover dermatology and ophthalmology consultations, are also being piloted within the DHP framework [37]. These services are currently mainly used in tertiary hospitals to support regional facilities. However, as the legal basis for teleconsultations has not yet been fully established, their uptake remains limited.

##### Lithuanian DHP Health Portals: Patient, Specialist, and Pharmacist Portals and Mobile App

DHP uses a set of web portals to accommodate its diverse user base, ensuring functionality for patients, health care professionals, and administrators. Lithuanian DHP provides a free-of-charge specialist portal (a portal intended for licensed health care professionals, including both general practitioners and specialist physicians), which has basic EHR functions and provides clinicians access to patient data as well as functions to manage patient records.

The platform’s portals are categorized as follows: (1) public informational portal [14]: provides general information about the eHealth system and its digital services to the public; (2) patient portal: allows Lithuanian citizens and residents to access their personal health records, including visit summaries, prescriptions, test results, and referrals; (3) specialist portal: enables health care professionals to view and manage patient records, submit clinical documents, and issue ePrescriptions and referrals; (4) pharmacist portal: allows pharmacy staff to access ePrescriptions and manage their dispensation. While pharmacists cannot alter the prescription itself, they are responsible for verifying and recording the issuance of prescribed medications; and (5) administrator portal: supports administrative tasks such as user account management, access control, and system configuration. In addition, the appointment booking system is implemented as an independent system, integrated with the DHP.

In March 2025, an official mobile app was released, providing authenticated access to patient personal records (visit history, ePrescriptions, and referrals/certificates), as well as allowing appointment booking and reminders. The app is designed to complement the web patient portal as a more convenient consumer interface to the DHP [50].

The portals and mobile app support multiple user authentication methods, including national electronic identification (eID) services, bank-based authentication, and mobile or digital signature solutions provided through the national eGovernment infrastructure.

##### DHP Subsystems

In addition to its core repository of clinical documents, the DHP also incorporates several dedicated subsystems that provide specific functionalities. These subsystems represent extensions of the platform rather than its entirety, and they currently cover only selected domains of care. The following description focuses on selected subsystems that are most relevant for illustrating core platform functionality and national-level integration, while other components are summarized in Table 2.

The ePrescription subsystem facilitates the electronic issuance, centralized storage, and [51] management of prescriptions, including those for extemporaneous medications, reimbursable pharmaceuticals, and medical devices [48]. This subsystem enables physicians to create and sign ePrescriptions that are securely stored and accessible to pharmacies for dispensing. Pharmacists can retrieve these prescriptions, dispense medications or devices, and update the system with information about the issued items via DHP’s pharmacist portal or using private solutions interfaced with the DHP. Patients using the patient portal can access their ePrescription history, view details of prescribed and dispensed medications or devices, and track their usage. Today, all reimbursed medicines are prescribed via ePrescription [51].

The national PACS subsystem, called “MedVAIS,” is envisioned as Lithuania’s national system for medical image archiving and exchange. While it aims to enable electronic submission, long-term storage, and sharing of diagnostic images among health care institutions and professionals [49], its practical implementation is limited, and its modernization is ongoing. Currently, the DHP primarily stores radiologists’ reports in document form rather than actual medical images (Figure 3). National PACS is meant to support the idea of reducing duplicate diagnostic procedures and fostering telemedicine capabilities. The infrastructure is integrated with health care institutions’ HIS/radiology information systems but does not yet fully realize its potential for image storage or exchange. This gap limits its ability to significantly enhance care coordination, although the foundational system provides the basis for future developments in digital health services.

National Remote Purchasing of Prescription Medications System (NPRVP), launched in 2023, enables patients in Lithuania to purchase prescription medications and reimbursed medical aids online. Integrated with the ePrescription, it allows users to compare prices, copayments, and delivery options across participating pharmacies. Patients securely access the system using electronic signatures or online banking credentials, with data protection ensured through multilevel encryption. NPRVP is aimed at improving access for individuals with, for example, mobility challenges [52].

The electronic drug-drug interaction subsystem within the DHP automatically checks medication compatibility during prescription issuance via the ePrescription. This subsystem alerts providers to significant pharmacokinetic and pharmacodynamic interactions, offering recommendations and detailed analysis options. Covering around 20,000 interactions and updated quarterly with international clinical data, it minimizes risks of adverse effects from polypharmacy while enhancing patient safety and care quality [53].

In line with the 2023-2027 Action Plan for Digital Health System Development [37], Lithuania is undertaking a comprehensive modernization of its national eHealth infrastructure. A key element of this is the implementation of EU cross-border health data exchange via the National Contact Point, enabling the interoperability of ePrescriptions and patient summaries with multiple EU countries, including Latvia, Estonia, Poland, Spain, Greece, Portugal, and others [54]. Within the national DHP, several new subsystems are being integrated or upgraded. These include the electronic laboratory subsystem for the centralized management of structured laboratory reports, allowing exchange of laboratory orders, specimens, and diagnostic results [55].

According to the 2023-2027 action plan for digital health system development [37], Lithuanian DHP will integrate new functionalities such as new preventive programs and subsystems that will coordinate invitations, capture test results, and support quality monitoring [56]. For example, a “medical clusters” data platform will track stroke [57], myocardial infarction, oncology, intensive therapy, trauma, child delivery and newborns, and organ donation pathway performance and outcomes [58]; an emergency-care analytics layer will provide real-time views of emergency department capacity to optimize patient flow and dispatcher routing [59]. In parallel, telemedicine and imaging exchange functions are subject to modernization until 2026 [60,61].

##### Appointment Booking System

The appointment booking system (*Išankstinė pacientų registracija* [IPR]) digitalizes outpatient appointment scheduling, allowing patients to book, cancel, and manage appointments online, view physician availability, and receive reminders on a national level. Integrated with the DHP, it supports services requiring electronic referrals and enables parents to schedule appointments for their children [62]. Before IPR, appointments were managed separately by each provider, largely by phone, with heterogeneous rules and limited visibility beyond the local facility. A single national entry point therefore standardizes booking steps (including electronic referral validation for services that require referrals), exposes country-wide slot availability, and lets patients switch to the earliest suitable specialist or location, reducing geographic inequities [62,63]. Queue enrollment and automatic notifications for earlier openings help backfill cancellations and shorten effective waits, while reminders and previsit prompts reduce no-shows [63]. By standardizing appointment workflows and exposing slot availability, IPR supports queue management and more equitable access to qualified specialists, shorter waits, and greater transparency.

Use has risen markedly in recent years (Figure 3 and Table 1). Over 3 months, in primary care, 298 out of 460 institutions used IPR; 2,149,678 (42.6%) out of 5,049,063 appointments reimbursed by the NHIF were registered via IPR [39]. However, only 5.7% of bookings were created by patients themselves; others were done by health providers (data from May 2025). The situation is relatively similar in specialist care, where over the last 3 months, 365 out of 584 institutions used IPR; 1,678,699 out of 2,219,583 appointments were registered via IPR, with 532,592 provider-created and 64,741 (10.8%) patient-created bookings in May 2025 [39].

**Figure 5:**
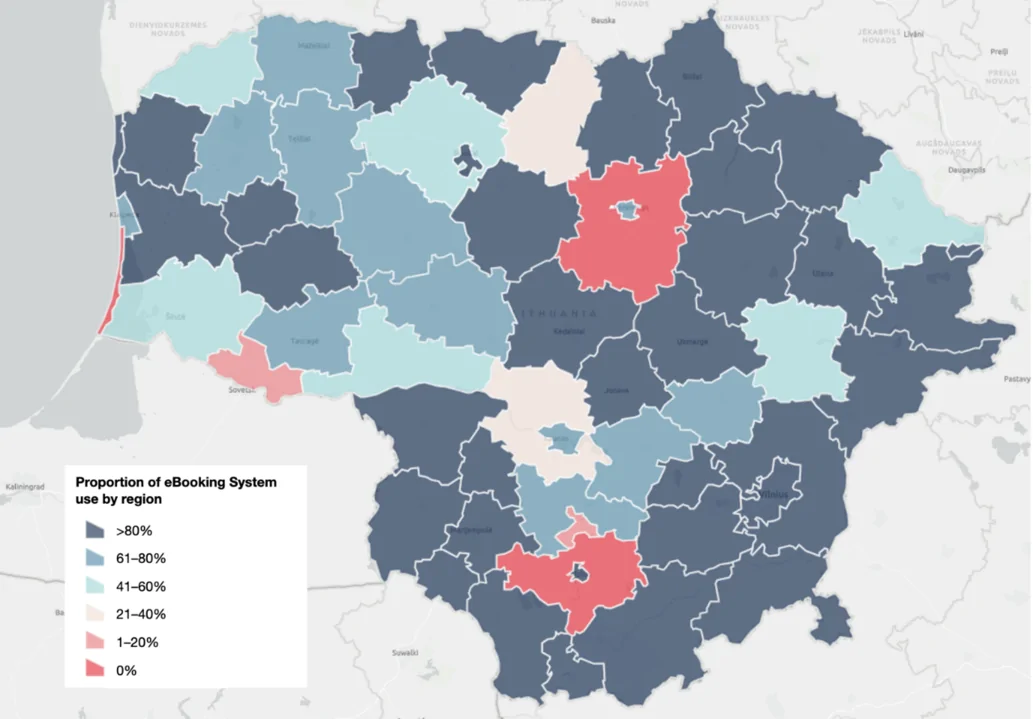
Appointment booking system coverage for specialist bookings by county in Lithuania.

Relatively low overall percentage of usage of this system could be attributed to uneven coverage across the country, with higher uptake in larger urban regions such as Vilnius County (69.4% of primary care visits booked via IPR) compared to lower rates in peripheral counties (Figure 5 [39]).

#### Other Registers and Monitoring Systems

Lithuania’s digital health is supported by a range of national registers and monitoring systems administered by different institutions (Table 2). Notably, the State Medicines Control Agency operates a register for pharmaceutical licensing, the authorized medicines register, and pharmacovigilance reporting, while the Institute of Hygiene maintains core population-health registers and surveillance systems used for epidemiology and service planning. These datasets interoperate with the DHP via standard state identifiers (eg, population and address registers), enabling consistent patient matching and enrichment of clinical records. However, their integration is uneven: while some registries directly support DHP operations, others remain linked only through one-directional data flows, for example, from DHP to the Institute of Hygiene for statistical processing, without returning to patient-facing systems.

### Health Information Exchange: Interoperability Standards and Security

Data sharing and interoperability within Lithuania’s digital health architecture are implemented using internationally recognized standards and defined national interface specifications. HL7 FHIR provides the API-led framework for creating, validating, and exchanging clinical resources across heterogeneous HIS and the national platform [64]. Nationally mandated terminologies (Systematized Nomenclature of Medicine-Clinical Terms [SNOMED CT] for clinical concepts and LOINC for laboratory observations) standardize content and enable consistent decision support and analytics across institutions. Profiled FHIR implementation guides and conformance testing are planned to underpin subsystem integrations to ensure predictable behavior of message payloads and document templates.

Enhancing semantic interoperability, the openEHR standard is being introduced in selected domains to strengthen semantic consistency through archetypes and templates that formalize the structure and meaning of clinical data across systems [65]. For secondary use, in pilot projects, selected priority datasets are being mapped to the Observational Medical Outcomes Partnership Common Data Model, enabling linkage and federated analyses with international observational research networks while preserving provenance and versioning of the original clinical data [66].

Security and trust are enforced through the state secure network and trust services operated by the Core Centre of State Telecommunications [67]. Communications between provider HIS and the DHP use mutually authenticated channels with Transport Layer Security 1.2/1.3. System components and users are identified via qualified certificates, and clinical documents with legal effect are sealed using qualified electronic signatures backed by hardware security modules. Comprehensive audit trails, tamper-evident logs and continuous monitoring support accountability, while data minimization, purpose limitation, and retention controls align with GDPR and national data-protection law.

From a health care practitioner perspective, authentication binds the user’s identity to their professional license by the national identification number before granting access to clinical information systems; authorization then constrains scope according to role, specialty, and organizational assignment, enforcing least-privilege access and ensuring data are processed only for legitimate care purposes [68]. Named-user credentials, session management, and auditability at the individual-account level enable post hoc review and sanctioning of misuse.

In the DHP, patient identity verification is performed through officially supported eID methods (mobile qualified e-signature, qualified e-signature on smartcard/USB token, bank-based authentication, and national electronic ID). After successful authentication, patients access defined electronic services and can view access logs; delegated access allows authorized proxies (eg, parents, carers, and legal representatives) to act within granted permissions. This puts Lithuania among the 8 EU member states scoring full points in accessibility within the Digital Decade Policy Program 2030 report [9].

## Discussion

### Overview

This study provides a comprehensive overview of Lithuania’s national digital health system, tracing its development over more than two decades and identifying its core components, governance mechanisms, and ongoing challenges. We argue that Lithuania has built a centralized yet multilayered infrastructure that integrates local HIS with national platforms and registers. The system has achieved high adoption of EHR and ePrescriptions, supported by a strong legal and technical framework. However, challenges remain in enhancing interoperability, modernizing underdeveloped subsystems, and increasing public and professional engagement with digital health tools.

In a broader European context, Lithuania’s centralized digital health architecture represents one of several approaches to national health information exchange. Across the WHO European Region, countries have adopted both centralized and decentralized models, with variation in governance, interoperability, and system maturity [69]. While centralized platforms can facilitate standardization, integration, and rapid national-scale deployment, they are also associated with challenges related to system complexity, flexibility, and user adoption. Conversely, decentralized or hybrid models may better accommodate local workflows but often face interoperability and coordination challenges. Countries with more mature systems, such as Estonia and Australia, tend to provide detailed operational frameworks and clearly defined institutional responsibilities, whereas others exhibit more fragmented or less structured approaches [70].

The Lithuanian experience suggests that the primary advantage of a centralized model may not be the existence of a single technical platform itself, but rather the ability to establish common governance mechanisms, legal requirements, and interoperability standards across the entire health system. Nationwide adoption of core services such as ePrescriptions, referrals, and clinical documentation was achieved through a combination of centralized infrastructure and mandatory participation. At the same time, the Lithuanian case demonstrates that technical interoperability and large-scale data collection do not automatically translate into high-quality, reusable clinical data or optimal user experience. Persistent challenges related to usability, workflow integration, data quality, and long-term system evolution indicate that governance, financing, stakeholder engagement, and implementation capacity may be as important as architectural design. These findings suggest that countries considering centralized digital health strategies should view technical integration as only one component of a broader transformation process requiring sustained institutional commitment and continuous system improvement [71,72].

Lithuania’s centralized governance model enabled rapid scale-up and national coverage. Mandating use of the DHP together with technical standards and secure infrastructure has supported consistency and compliance. Available reports and evaluations indicate usability and performance challenges with central DHP portals, including complex workflows, duplicate data entry, slow system response, and periodic instability, which may disrupt clinical workflows and contribute to continued reliance on local EHR and HIS solutions [32]. In addition, an informal survey of health care professionals (n=884) conducted by the Lithuanian Medical Movement reported low satisfaction of health care professionals with usability of digital health systems in Lithuania, with 75.3% of respondents rating the digital systems as “inconvenient” or “very inconvenient,” and 47.4% reporting poor access to patient data [73]. However, the survey’s methodology does not allow clear attribution of this dissatisfaction to either the national DHP solution or local HIS solutions. While such findings suggest significant usability concerns, systematically published evidence on clinician experience remains limited, highlighting the need for further empirical research.

Functionality gaps (such as imaging exchange and telemedicine), uneven interoperability within the system and with EU partners, and variable awareness and use among patients and clinicians indicate the need for continued user-centered improvement, stakeholder engagement, and technical renewal. From this experience, we identified five areas for discussion: (1) governance and continuity, (2) institutional competence and delivery capacity, (3) technical architecture and interoperability, (4) financial sustainability, and (5) meaningful use of structured data, which represent the main challenges encountered during the development of Lithuania’s digital health system.

### Governance and Continuity

Ensuring policy consistency and continuity was a persistent challenge in the evolution of Lithuania’s national digital health system. Across successive government terms, priorities and delivery plans were reset as administrations changed, making a stable direction in both health policy and digitalization difficult to maintain. The national digital platform and its major programs are publicly financed (state budget with EU cofinancing) and governed by state institutions (the Ministry of Health as policy owner and SECR as system operator), so program direction has tracked administrative cycles. Due to the government changes linked to the political cycles, external reviews conducted by universities and the National Audit Office recorded timetable delays, scope changes, and incomplete delivery against stated agendas [32,74].

Comparable challenges are reported internationally. Evaluations of large, centrally led programs describe how shifts in strategy, reorganization, and limited stakeholder engagement have been followed by delays, cost growth, and uneven adoption. The English National Program for IT is a common example [75,76]. Implementation reviews emphasize that uptake is most influenced by governance, clear decision rights, user-centered design, and organizational readiness rather than political branding [77,78]. Comparative policy reports also mention these points, highlighting leadership, data governance and sustained investment as prerequisites for system-level benefits [79]. EU guidance likewise stresses stable governance and interoperability as the basis for cross-border digital services [80] and calls for multiyear strategies aligned to resources [81].

Implementation research and comparative reporting indicate that coordination is most effective when digital tools are designed around clinical workflows, interoperable standards and organizational readiness, and when trust and transparency are maintained [77-79,81]. Although a stakeholder platform created within a research project was not sustained as a policy instrument [74], several arrangements appear to have benefited delivery despite administrative turnover. Formal instruments, such as multiyear strategies and legal acts, provided continuity and clarified decision rights. Portfolio coordination between the Ministry of Health and the eHealth Development Coordination Board through scheduled meetings, structured reporting and investment planning helped maintain alignment.

### Competence and Capacity

The lack of competence in health care digital transformation, particularly when internal capacity stagnates due to overreliance on outsourcing to private entities, has emerged as a critical issue in both academic and policy discussions. This dynamic raises concerns about institutional sustainability, innovation control, and digital sovereignty. Government and public health institutions often pursue rapid development of digital health systems to improve efficiency and patient outcomes. However, many lack sufficient in-house expertise, relying instead on private vendors for technical development. Outsourcing addresses immediate gaps but can produce long-term delays in internal competencies essential for strategic control and sustainable innovation.

Comparable patterns are reported internationally. Studies argue that overreliance on outsourcing in public IT projects leads to a “hollowing out” of capabilities [75,82], creating a vicious cycle: the less internal knowledge, the greater the dependency on vendors, further weakening public innovation capacity. Outsourcing may provide speed and technical sophistication, but it shifts strategic control to private actors, risks vendor lock-in, and impairs governments’ ability to adapt systems to new needs or ensure public value [80]. Evaluations of large national programs in the United Kingdom have also shown that local actors often feel alienated when practical knowledge is ignored during planning, leading to weak adoption and costly delays [76,83]. Implementation research consistently highlights the importance of participatory, context-sensitive approaches that recognize local knowledge and build trust [77,78].

In Lithuania, similar challenges have been reported in national evaluations and policy analyses. Previous audits and assessments have highlighted fragmented governance, limited continuity of institutional knowledge, and reliance on project-based implementation models, which may hinder the accumulation of internal expertise and long-term system development [32]. Frequent contractor changes and limited mechanisms for knowledge transfer have been identified as contributing factors. In addition, the relatively small pool of qualified professionals may constrain institutional capacity and continuity. These dynamics may weaken strategic control over system development and contribute to governance fragmentation, particularly when national initiatives are implemented in a top-down manner without sufficient alignment with local infrastructure and clinical practice [32].

Workforce education and training remain an important but relatively underdeveloped component of digital health capacity in Lithuania. Training activities do exist, including instructional materials, video tutorials, and institution-level training sessions provided through the national eHealth portal and related initiatives [84]. However, these are primarily focused on system use and operational onboarding rather than structured, system-wide development of digital health, data governance, and health informatics competencies. Responsibility for workforce development is distributed across multiple institutions, including universities, professional bodies, and the Ministry of Health, without a single coordinating framework. While national strategies emphasize the importance of digital skills, mechanisms for systematic capacity building, long-term training programs, and dedicated funding remain limited. Strengthening coordinated workforce development will be essential to support sustainable digital health system evolution.

Several solutions have been proposed. International experience indicates that creating dedicated national competence centers can mitigate outsourcing dependency by consolidating expertise and ensuring continuity. Lithuania has not adopted such an approach. International methodological frameworks also emphasize structured governance and delivery capacity as prerequisites for sustainable digital health development [29]. Stakeholder engagement is also crucial. Involving patients, caregivers, clinicians, IT developers, policymakers, data protection bodies, researchers and nongovernmental organizations is consistently emphasized as a cornerstone of effective digital health development. In Lithuania, a stakeholder discussion platform was piloted through a research project using collective intelligence methods [74], but its management at the national level was not sustained. More durable solutions require the establishment of joint task forces and multistakeholder boards composed of local and national representatives, supported by empowered local digital leaders liaising directly with national authorities. The Lithuanian arrangement of an eHealth Development Coordination Board overseeing financing and development decisions provided some continuity, but its impact was limited, underscoring the need for stronger, sustained institutional mechanisms.

### Technology and Interoperability

Both IT and interoperability standards evolve, which brings additional challenges on the level of the national DHP to keep up with the technology and normative versions of the standards. From the DHP initialization, its underlying standard, HL7 FHIR, has evolved dramatically from the initial 0.8 version to a mature 5.0 release. At the same time, globally competing clinical data modelling and data exchange standards started converging. Namely, initiatives of mutual use of HL7 FHIR and openEHR are already being discussed [85,86]. These developments raise conceptual and technically complex questions on the transition, migration, or keeping multistandard and multiversion clinical data in the unified national EHR.

Lithuania was an early adopter of HL7 FHIR, which facilitated the rapid development of a nationwide health information exchange. However, after nearly a decade of operation, elements of the DHP can be characterized as legacy systems, in the sense that parts of the architecture rely on earlier design choices that limit flexibility, scalability, and integration with newer technologies. This has led to the accumulation of “technical debt,” defined here as the need for additional development effort to address architectural constraints, maintain system performance, and enable further evolution.

Evidence from national evaluations and audit reports points to recurring issues such as incomplete functionality, integration challenges, and the need for ongoing system modernization, which are consistent with the presence of technical debt [32]. These factors suggest that continued investment in system maintenance and architectural renewal is required to ensure long-term sustainability.

The main lessons learned are to ensure continuous effort in maintenance and upgrade of the technology created to minimize the technical debt. Moreover, it is important to prioritize the DHP architecture over tactically important features, and to follow the reference DHP architecture [68], whenever possible. Moreover, data governance should be exercised systematically, covering the full cycle starting from planning, analysis, design, monitoring, evaluation, transition, and decommissioning. Dedicated roles with responsibilities for data governance shall be established.

### Financial Sustainability

Sustainable financing was a key constraint. Across development phases, funding has been provided through a combination of EU structural funds (primarily supporting capital investments), national budget cofinancing, and institution-level resources, with operational components often delivered through vendor contracts. However, financing has remained largely project-based, with limited dedicated funding for long-term maintenance and system evolution. This has contributed to the accumulation of technical debt and repeated replanning.

This pattern was flagged by the National Audit Office in 2017, which urged the MoH to ensure a durable governance and financing model before the next development phase [32]. Budgetary capacity is also limited in comparative terms: total health spending was 7.3% of GDP in 2023, well below the EU average (10%), limiting room for recurrent digital budgets unless explicitly allocated [7]. International evidence underscores that underinvestment in technology maintenance is common. European analyses have repeatedly noted that IT investment in health care providers remains modest relative to overall operating budgets. EU-level evaluations have also warned that fragmented and short-term funding models undermine the sustainability of digital health programs and prevent the full realization of expected benefits [79,80].

Taken together, the Lithuanian experience shows that financial sustainability is critical for program continuity. Moving from project-based investment to whole-life financing with multiyear operations and maintenance (hosting, licenses, cybersecurity, monitoring, 24/7 support, and messaging) and stable product teams for core platforms should reduce technical debt and stabilize delivery. Modular procurement based on open standards, combined with independent testing, helps avoid dependence on single vendors and keeps costs under control. Consistent with the WHO eHealth Strategy Toolkit’s requirement to estimate and time financing needs within the action plan [81], and World Bank guidance that countries should establish a budget line item for digital health [87], a recurrent, scheduled appropriation rather than ad hoc project funding would greatly improve the financial sustainability of the national digital health system.

### Meaningful Use of Structured Data

Although the national platform stores structured data, use at the point of care is still largely document-level rather than granular—data item level. Many integrations use signed clinical summaries or other patient documents that clinicians can view but cannot easily filter, search, or reuse inside their local HIS or the DHP specialist portal, so decision support and cross-episode review remain limited. Data models and coding are uneven across sources: templates have evolved at different times, and some external registers apply alternative code systems or value sets, so the same concept may be represented differently in the DHP versus other national registers, leading to inconsistent counts and analytics. Change control has been slow and rigid: adding or revising data elements often requires creating a new template rather than reusing an existing one, so providers adopt local workarounds and duplication persists. Modernization is planned to address these issues with a DHP decomposition project, aimed at clearer conformance rules, updated interfaces and template governance [88], but until implemented at scale, depth of structured exchange and reuse will remain constrained. In the long-term, AI-based methods could also be implemented to support the conversion of unstructured clinical narratives into structured data, reducing manual effort and improving data reusability.

### Future Developments

The next phase in Lithuanian digital health development focuses on decomposing and stabilizing the core platform and tightening interoperability while enabling analytics and responsible innovation. The action plan 2023-2027 [37], supported by the Recovery and Resilience Facility–funded projects [89], involves updating and validating minimum HIS integration requirements and introducing real-time performance monitoring and stronger user support—steps intended to reduce technical debt and improve usability without adding new central obligations. Stated priorities include renewing the national PACS for standardized imaging exchange, implementation of telemedicine services for selected specialties, and development of new analytical subsystems, such as preventive program coordination, clinical cluster monitoring, and an emergency care analytics layer to support real-time capacity management. Collectively, these initiatives seek to unify definitions, strengthen data governance, and establish conditions for value-based care and secondary data reuse.

Recent national policy guidelines project outline a structured roadmap for the integration of AI into the Lithuanian health system for 2026-2031 [90]. These plans include the development of a national health data architecture and a unified clinical data model to ensure that all clinically relevant data are structured, standardized, and suitable for machine processing. Additional measures include the creation of a national AI platform integrated with existing systems (eg, DHP and PACS), the establishment of a certified AI solutions catalog, and the implementation of regulatory frameworks for testing, validation, and monitoring of AI systems in line with EU legislation [90].

Planned use cases include clinical decision support, medical imaging analysis, population health analytics, and patient-facing digital tools supporting self-management and preventive care. The strategy also foresees the development of secure data access mechanisms (eg, “data marketplace” models), expansion of real-world data use for research, and implementation of AI-assisted data entry tools to improve data quality. Workforce development is addressed through dedicated training programs for clinicians and policymakers, while innovation is supported through regulatory sandboxes enabling controlled testing of AI solutions in clinical environments [90].

Implementation is planned in 3 phases: an initial preparation phase (2026-2027) focusing on data architecture and pilot projects; a system transformation phase (2028-2029) involving deployment of AI platforms and integration into clinical workflows; and a scaling phase (2030-2031) aimed at nationwide adoption. While these initiatives position Lithuania for AI-enabled health care, their success will depend on data quality, governance capacity, and the ability of the existing DHP infrastructure to support scalable and interoperable solutions.

### Study Limitations

This study relied solely on publicly available documents, which primarily reflect official and institutional perspectives. This approach may not fully capture the experiences, opinions, or challenges faced by health care professionals, patients, or private-sector stakeholders. The analysis focused on national-level initiatives and does not address variation in implementation and use across different regions, institutions, or clinical domains. Document analysis has inherent constraints: the availability and accessibility of materials may be selective; documents can frame policy processes in ways that emphasize achievements while downplaying shortcomings, and interpretation requires subjective judgment, even with systematic review methods. We did not perform interviews or surveys, and public user satisfaction data is limited, which could have provided richer insights into stakeholder perspectives and practical implementation dynamics.

Moreover, several of the authors have previously held formal roles in the Ministry of Health or the National eHealth Development Coordination Board. While this insider perspective informed contextual interpretation, it may also introduce bias toward institutional viewpoints.

Finally, the study does not include quantitative outcome measures (eg, cost-effectiveness, clinical impact, and patient experience) and therefore cannot directly evaluate the performance of the Lithuanian digital health system. Instead, it provides a descriptive and interpretative account of governance structures, infrastructure, and challenges, which should be complemented by future empirical research.

### Conclusion

Lithuania’s digital health architecture illustrates how a small country can develop a complex yet functional national system. A clear legal and institutional framework has provided authority and consistency in governance, while the integration of multiple national registers and adoption of international standards such as HL7 FHIR signal a strong commitment to interoperability and scalability. The SDA’s model of collecting and managing health data for secondary use, rather than pursuing direct integration, represents an alternative pathway for leveraging information in policymaking, research, and system optimization.

At the same time, the system’s heavy reliance on top-down governance has limited flexibility and stakeholder autonomy, and the slower progress of certain subsystems highlights the challenge of sustaining momentum and investment over time. Persistent issues include achieving full interoperability, strengthening interinstitutional collaboration, and modernizing underdeveloped components.

Lithuania’s experience demonstrates both the opportunities and constraints of centralized digital health governance. For other countries, it highlights that centralized governance can accelerate nationwide adoption of digital health services, but long-term value depends on sustained investment, stakeholder engagement, data quality improvement, and the ability to adapt infrastructure to evolving clinical and technological needs.

## Data Availability

The study is based entirely on publicly available documents and official statistics. All data sources are referenced in the manuscript, and no additional data are available beyond those cited.

## Abbreviations

DHP: digital health platform
EHDS: European Health Data Space
EHR: electronic health record
eID: electronic identification
ePrescription: electronic prescription
ESPBI IS: Electronic Health Services and Cooperation Infrastructure Information System (Elektroninė sveikatos paslaugų ir bendradarbiavimo infrastruktūros informacinė sistema)
ESPBI: Elektroninė sveikatos paslaugų ir bendradarbiavimo infrastruktūros
EU: European Union
epSOS: Smart Open Services for European Patients
FHIR: Fast Healthcare Interoperability Resources
GDP: gross domestic product
GDPR: General Data Protection Regulation
HIS: hospital information system
HL7: Health Level 7
IHE: Integrating the Healthcare Enterprise
IPR: Išankstinė pacientų registracija
LOINC: Logical Observation Identifiers Names and Codes
NESS: National eHealth Service System
NHIF: National Health Insurance Fund
NPRVP: National Remote Purchasing of Prescription Medications System
PACS: picture archiving and communication systems
READ: readying, extracting, analyzing, and distilling
SDA: state data agency
SECR: State Enterprise Centre of Registers
SNOMED CT: Systematized Nomenclature of Medicine-Clinical Terms
SVEIDRA: Lithuanian title of a National Health Insurance System
WHO: World Health Organization
